# Stability Study of Graphene Oxide-Bovine Serum Albumin Dispersions

**DOI:** 10.3390/jox13010008

**Published:** 2023-02-16

**Authors:** Javier Pérez-Piñeiro, Fernando Sánchez-Cea, Mariana P. Arce, Isabel Lado-Touriño, María Luisa Rojas-Cervantes, María Fuencisla Gilsanz, Darío Gallach-Pérez, Rodrigo Blasco, Niurka Barrios-Bermúdez, Arisbel Cerpa-Naranjo

**Affiliations:** 1 School of Architecture, Engineering and Design, European University of Madrid, C/Tajo s/n Villaviciosa de Odón, 28670 Madrid, Spain; 2 Department of Inorganic and Technical Chemistry, Universidad Nacional de Educación a Distancia (UNED), Urbanización Monterrozas, Las Rozas, 28232 Madrid, Spain

**Keywords:** graphene oxide, biologic fluids, bovine serum albumin

## Abstract

In this work, a stability study of dispersions of graphene oxide and graphene oxide functionalized with polyethylene glycol (PEG) in the presence of bovine serum albumin is carried out. First, a structural characterization of these nanomaterials is performed by scanning electron microscopy, atomic force microscopy, and ultraviolet visible spectroscopy, comparing the starting nanomaterials with the nanomaterials in contact with the biological material, i.e., bovine fetal serum. The different experiments were performed at different concentrations of nanomaterial (0.125–0.5 mg/mL) and BSA (0.01–0.04 mg/mL), at different incubation times (5–360 min), with and without PEG, and at different temperatures (25–40 °C). The SEM results show that BSA is adsorbed on the surface of the graphene oxide nanomaterial. Using UV-Vis spectrophotometry, the characteristic absorption peaks of BSA are observed at 210 and 280 nm, corroborating that the protein has been adsorbed. When the time increases, the BSA protein can be detached from the nanomaterial due to a desorption process. The stability of the dispersions is reached at a pH between 7 and 9. The dispersions behave like a Newtonian fluid with viscosity values between 1.1 and 1.5 mPa·s at a temperature range of 25 to 40 °C. The viscosity values decrease as the temperature increases.

## 1. Introduction

Nanotechnology has had a great impact in the last two decades as it offers different properties to volumetric materials. One of these nanomaterials is graphene oxide (GO), which can be obtained from graphite. Graphite can be exposed to strong oxidants that produce graphite oxide. This can be exfoliated through an acid-base treatment, producing planar monomolecular layers, which are graphene oxide [1].

Graphene is a nanomaterial consisting of a single layer of allotropically organized carbon atoms from graphite. Each layer is composed of lattices divided into two sublattices connected by σ bonds, in which each carbon possesses free π electrons. These bonds create a high electron density both over and under the 2D layer of graphene [2].

It is a chemically, mechanically, and thermally stable material and possesses high flexibility. Graphene has a distinct structure that confers special properties, such as a large specific area, the ability to be easily modified, and the fact that it is a long-lasting material. These properties enable graphene to act as a drug delivery vehicle [3].

Graphene-based nanomaterials exhibit characteristics that make them very versatile and useful in a wide variety of fields, including biomedical applications, fast-charging batteries, advanced environmental photocatalysts, solar cells, and quantum physical chemistry, among others [4].

They acquired notable attention in the field of biomedicine, such as in tissue engineering for tissue repair, regeneration of bone structures, and drug delivery [5]. For example, oxygen-poor graphene particles are used for osteo-induction that aids bone regeneration [6].

Graphene nanomaterials have the ability to bind to DNA and protect it from nucleases, enzymes that degrade nucleic acids. This property has been investigated for its use in gene therapy techniques for “gene targeting”. Because it protects the DNA and promotes more efficient and easier gene fragment insertion, it allows us to ensure that the insert reaches the target site [2].

The unique structure of graphene, as well as its C-C bonds, provide it with excellent thermal and electrical conductivity that can be useful in electronic and biomedical instruments such as biosensors [7]. A biosensor is an instrument with a biological component, a transducer, and a detector that measures certain parameters. They can be used to enhance the properties of both enzymatic and non-enzymatic biosensors: glucose sensors, hydrogen peroxide sensors, and immunosensors [8].

GO presents a great variety of functional groups such as oxygens, epoxide groups, carbonyl, hydroxyl, and phenol groups, which are distributed along the GO surface [2]. The most apparent difference compared to graphene is the presence of oxygen atoms attached to carbon atoms [9]. Its basal structure of graphene provides it with many free π electrons which are hydrophobic and allow π-π interactions. It in turn possesses both aromatic (sp^2^) and aliphatic (sp^3^) domains, which facilitate interactions at the molecular surface and can form hydrogen bridges and metal ionic bonds [2,9].

GO possesses properties that make it a perfect candidate for cancer therapy, using it as a drug transporter (drug delivery), as by modifying it, it can deliver the drug to its target more effectively [5].

Surface properties determine the properties of the nanoparticles; therefore, surface modification of nanoparticles is a useful process to modify the properties of nanoparticles by adding or modifying functional groups to the surface of the NPs. Several studies have been conducted to see how we can obtain better performance or improvements through the formation of functionalized nanomaterials with other particles by surface modification [10,11,12,13].

PEG is a very useful biological reagent due to its minimal cytotoxicity, high biocompatibility, protein resistance, and water solubility. It has been shown that, thanks to these properties, when PEG conjugates are produced with other polymers, an increase in biocompatibility is promoted [10].

In the same way, the surface PEGylation of graphene NPs has been extensively studied, concluding that it can have a good use as a hydrophobic anticancer drug carrier in mammalian organisms [10,12,14].

The usefulness of NPs in biomedicine is conditioned by their behavior in biological media. The study of nanoparticle-protein interactions is essential since this will help us understand the feasibility of their use in living organisms, opening the way to new possibilities in the biomedical field.

NPs are instantly modified once they come into contact with the bloodstream due to interactions with blood components. This spontaneous coating of NPs by proteins has been termed “protein corona” (PC) and is a determining factor for the pharmacology and toxicology of NPs [15].

The first proteins that form the PC bind to the NPs very quickly because of the affinity of their amino acids and side chains for the NPs. These proteins already anchored to the surface bind, but instead of binding to the surface of the NPs, this second layer of the PC is formed by protein-protein interactions. The binding of the protein to a nanoparticle forms the complex known as NP-PC, and the characteristics of both are modified in such a way that researchers have recently begun to treat them as new NPs [16].

During adsorption to NPs, proteins may undergo conformational modifications to rearrange their structure. Binding proteins to flat-surface NPs can lead to more abrupt structural changes in the proteins than if the NPs were curved surface. This process is thermodynamically favorable if the charged or hydrophobic sequences of the proteins coincide with the hydrophobic or charged surface portions of the NPs [17].

The affinity competition between proteins to bind to the surface of NPs is responsible for the protein corona changing over time; this allows us to distinguish between “hard corona” and “soft corona”. The “hard corona” is considered the first protein layer that binds tightly to the NPs and is maintained for a long residence time (several hours). The “soft corona” is represented by the second protein layer, which is not directly bound to the NP and undergoes very rapid changes in short periods of time. The proteins that are bound to the NP are in a continuous desorption/adsorption flux, mainly controlled by the so-called “Vroman effect” [16].

The composition of PC is unique for each nanomaterial and depends on its size, shape, and surface charge, as well as any surface modifications that it has and the biological environment in which it is found. Even when compared between NPs of the same material, it can vary, as it is highly dependent on the physicochemical properties of the NPs and the concentration of proteins present in the biological medium [17,18].

In this work, a stability study of graphene oxide and graphene oxide functionalized with polyethylene glycol (PEG) in the presence of bovine serum albumin (BSA) is carried out. Serum albumin is the most abundant protein found in blood plasma, constituting most of the plasmid fluid and playing an important role in the transport of compounds [19]. As such, it is necessary to deepen our understanding of the transport properties of these materials in biological fluids. Structural characterization of these nanomaterials is performed by scanning electron microscopy, atomic force microscopy, and ultraviolet-visible spectroscopy. In addition, a rheological characterization was made at different concentrations of GO and GO+PEG (0.125–0.5 mg/mL) and BSA (0.01–0.04 mg/mL), at different incubation times (5–360 min), and at different temperatures (25–40 °C).

## 2. Materials and Methods

### 2.1. Materials

Graphene oxide (GO) was acquired by NanoInnova Technologies SL. This GO has a specific area of 20–35 m^2^/g and an average pore size of 12.8 nm.

Biological fluids, bovine serum albumin (BSA, USA), and fetal bovine serum (FBS, South America), were obtained for Sigma Aldrich (Saint Louis, MO, USA) and Gibco (Thermo Scientific, Paisley, UK), respectively.

4arm-PEG5K-NH_2_ and 1-ethyl-3-(3-dimethylaminopropyl)carbodiimidehypochlorite (EDC HCl) were purchased from Sigma-Aldrich (Madrid, Spain).

### 2.2. Synthesis of PEGylated Graphene Oxide

For the PEGylation process of the nanomaterials, the method described by Zhu et al. [14] was followed with slight modifications.

**Figure 1:**
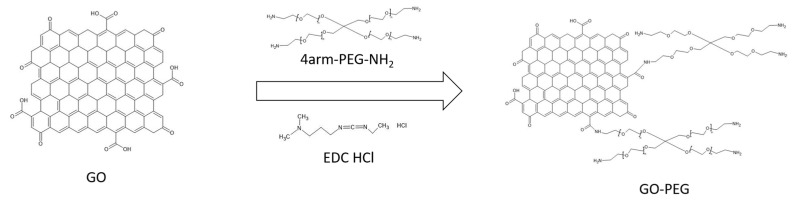
Scheme of the synthesis of graphene oxide pegylated.

EDC HCl is added to the nanomaterial dispersion, which is responsible for activating the carboxyl groups of the nanomaterial. This is achieved because the amide group of the EDC captures an H proton from the carboxyl group, and the EDC and the GO nanomaterial are joined, leaving an intermediate product called o-acylisourea on the surface of the nanomaterial. The PEG has branches and a terminal amino group. This amino will react with the o-acylisourea, breaking the R-COO-R bond and forming an amide bond between the nanoparticle’s R-COO- group and the PEG’s amino group, forming GO-PEG via an amide bond. The reaction leaves urea as a residue. PEGylation of GO gives us, for each PEG molecule, 3 functional terminal amino groups on the surface of the nanoparticle. A schematic of the reaction can be seen in Figure 1.

A dispersion of graphene nanomaterial of concentration 1 mg/mL (100 mg suspended in 100 mL of double-distilled water) is prepared, and then 4armPEG5K-NH_2_ (300 mg) is subsequently added. This mixture is sonicated in an ultrasonic bath for 5 min at room temperature. Afterwards, 30 mg of EDC HCl is added and sonicated in the ultrasonic bath for 40 min, after which EDC HCl (80 mg) is added again and left to stir overnight with magnetic stirring.

The dispersion was vacuum filtered and washed with water several times. The filtration result was placed in a crystallizer and allowed to air dry for 48 h at room temperature to remove any remaining moisture. It was obtained as a black solid that corresponds to GO-PEG.

### 2.3. Dispersions Preparation

Graphene oxide samples were mixed with a biological fluid to acquire suspensions with different concentrations. To obtain a high dispersion and homogenization, graphene oxide/graphene oxide pegylated dispersions were homogenized with an ultrasonic homogenizer (Bandelin, Germany) for 10 min using 50% power and a pulsed cycle of 1 s (active and passive intervals of 1 s). Then, the biological fluid was added, and an incubation was performed. Several incubation times (10 min, 30 min, 45 min, 60 min, 2 h, 4 h, and 6 h), different incubation temperatures (25, 30, 37, and 40 °C), and different concentrations (Table 1) were tested to check the influence of these parameters on the adsorption of bovine serum albumin on graphene oxide. The supernatant was removed after sedimentation to remove the possible excess of biological fluid and the molecules that were not linked to the nanomaterial.

### 2.4. Characterization

The characterization of GO powder and GO-FBS dispersion were performed by Cerpa et al. in previous papers using several techniques [10,20]. In this work, the structural characterization of graphene oxide, graphene oxide pegylated, and these nanomaterials with the biological fluid, bovine serum albumin, were performed.

Scanning electron microscopy images were captured using a JEOL JSM 6335F (JEOL Ltd., Tokyo, Japan) at 20 kV with a secondary electron detector in the Centro Nacional de Microscopía (Madrid, Spain). From a diluted suspension, one drop of each nanomaterial or nanomaterial with biological fluid was placed on a stainless-steel grid with a graphite layer and dried before analysis. Ultraviolet-visible spectra were obtained by the UV-Vis spectrophotometer Jasco V-730 (Madrid, Spain). Dispersions prepared in Section 2.3 were measured directly in UV-Vis equipment without previous preparation.

Measurements by zeta potential were carried out with a Zetasizer Nano ZS (Malvern, UK) instrument at pH values between 2.0 and 9.0. The zeta potential is connected to a MPT-2 Tritator (Malvern, UK) that perform the automatically pH regulation. A Haake RheoStress 6000 rotational rheometer (Thermo Scientific, Karlsruhe, Germany), equipped with a double-cone and plate system that has a 2° cone angle and a 60 mm diameter, was utilized to acquire the rheological properties of the samples. Each sample was measured with the same protocol of three stages. The first stage involves a linear increase in shear rate from 0 to 1000 s^−1^ in 300 s; the second stage is a plateau at 1000 s^−1^ for 60 s; and finally, the third stage decreases the shear rate from 1000 to 0 s^−1^ in 300 s.

## 3. Results and Discussion

### 3.1. Scanning Electron Microscopy Characterization

**Figure 2:**
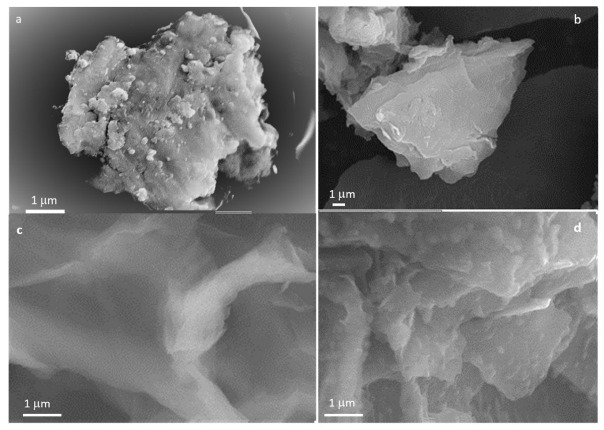
SEM images of (**a**) BSA, (**b**) GO, (**c**) GO-PEG, and (**d**) GO-PEG in BSA.

Structural characterization was performed by scanning electron microscopy to determine information about the size and shape of the nanostructures and test the possible morphological changes produced by the BSA interaction. Figure 2a–d shows the images of BSA, GO, GO-PEG and GO-PEG + BSA structures. The BSA has a shape similar to a cauliflower. Figure 2b,c show the images of GO and GO-PEG. These images present a sheet-like structure with a smooth surface, and in Figure 2d, the BSA is shown on the surface of GO-PEG.

### 3.2. Zeta Potential Measurements

These measurements provide quantitative information about the stability of the samples. A study of the zeta potential of GO + BSA was performed in a pH range of 2–9. Each sample was measured three times, and the average of these three measurements is shown in Table 2.

Different authors consider ±25 mV as a value for assuring the stability of suspensions [21]. It is concluded that graphene oxide with bovine serum albumin is stable at a pH = 7.4, which is the biological pH. Sanchez-Perez et al. determine that, at a fixed pH, it is the zeta potential that controls protein adsorption [22].

### 3.3. Ultraviolet-Visible Spectrometry Characterization

**Figure 3:**
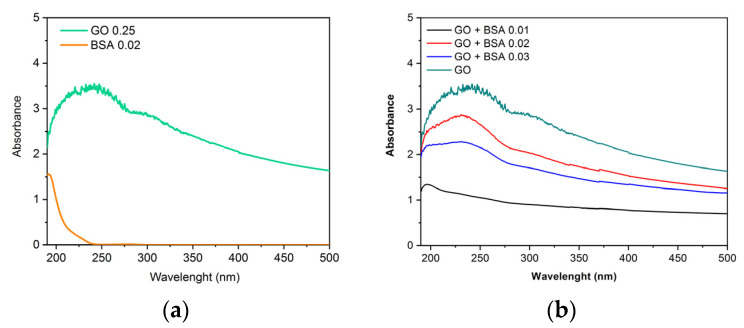
Absorbance vs. wavelength. (**a**) Spectra of GO (0.25 mg/mL) and BSA (0.02 mg/mL). (**b**) Spectra of GO (0.25 mg/mL) with different concentrations of BSA obtained by UV-Vis spectrophotometer.

Obtaining the spectra of the nanomaterials allows us to know their characteristic patterns, so it is possible to compare and observe the variations when the protein is added. In the spectrum corresponding to GO (Figure 3a,b), two peaks characteristic of this nanomaterial are observed. There is a maximum peak at 230 nm corresponding with the π-π* transitions of aromatic C-C bonds, and a shoulder near 300–310 nm assigned to the n-π* transitions of C-O bonds [23]. The BSA addition is not remarkable in Figure 3b.

In a previous work, Zhang H. et al. [24] demonstrated that the kinetics of BSA adsorption onto GO follows the pseudo-second-order kinetic model and that it is a quick process with high adsorption capacity. Some of the structure and functions of BSA were changed by GO binding. The spectra and modeling results indicated that the hydrophobic force, hydrogen bonds, van der Waals, and π-π* stacking interactions could contribute to the adsorption forces of BSA on the GO surface.

**Figure 4:**
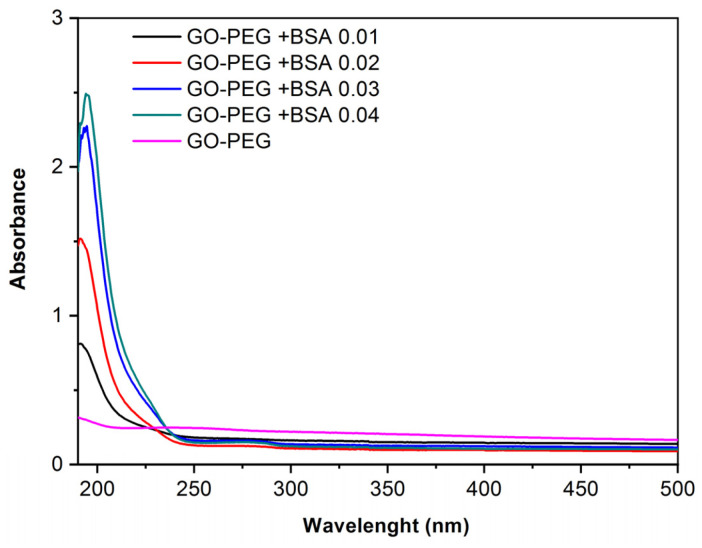
Absorbance vs. wavelength. Spectra of GO-PEG (0.125 mg/mL) and GO-PEG with different concentrations of BSA obtained by UV-Vis spectrophotometer.

However, in the spectra with GO-PEG and different concentrations of BSA (Figure 4), it is observed that a peak is formed around 190 nm, which, as concentration increases, tends to move towards 210–220 nm, corresponding to the main structure of albumin. The small absorption shoulder formed around 280 nm corresponds to aromatic amino acids [25,26]. This demonstrates that PEG improves the dispersion of the samples because in Figure 4 the characteristic peaks of BSA are observed, whereas in Figure 3 using GO without PEG it is not possible to observe these peaks.

**Figure 5:**
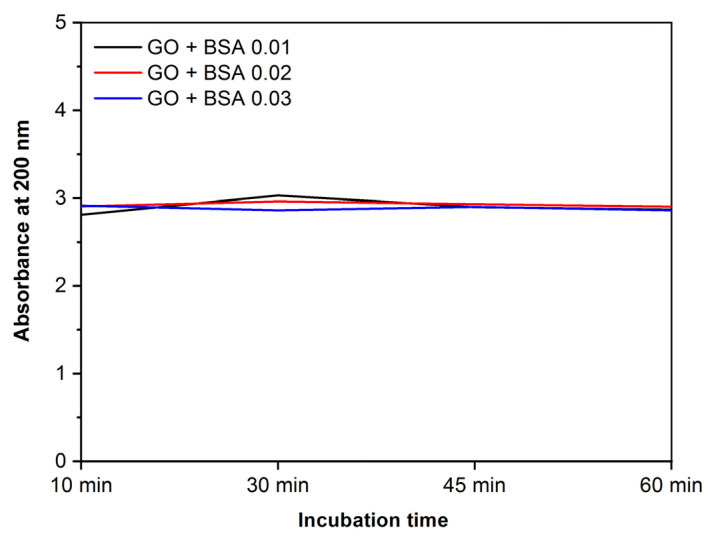
Absorbance at 200 nm vs. incubation time. GO (0.25 mg/mL) with different concentrations of BSA.

Samples containing GO and BSA at different concentrations (Figure 5) show that, in the first 60 min of incubation, the absorbance levels do not change. This shows that the desorption of the proteins bound to the nanoparticles does not happen in the first hour.

### 3.4. Rheological Studies

The analysis of the rheological results obtained allows one to check how the addition of nanomaterials affects the physical characteristics of biological fluids, specifically their flow behavior (flow curve) and viscosity values.

**Figure 6:**
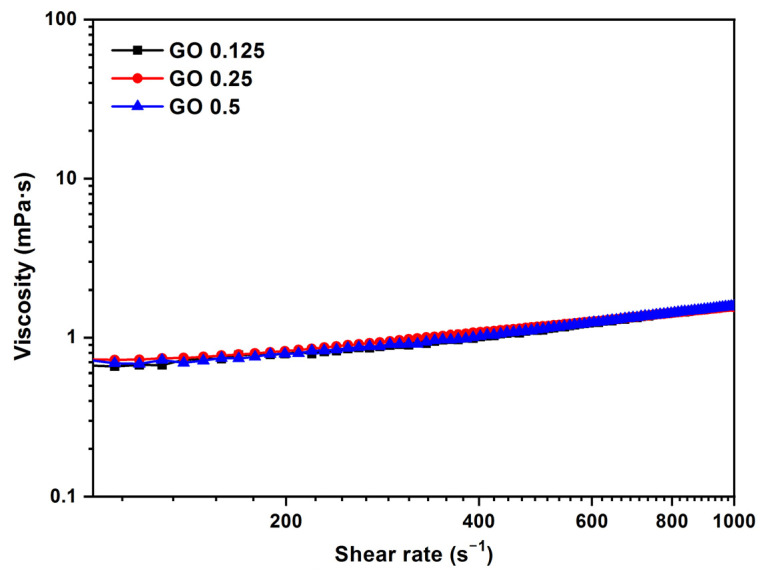
Viscosity (η) vs. shear rate ($\dot{\gamma }$) for different concentrations of GO (0.125, 0.25, and 0.5 mg/mL). Temperature: 37 °C.

The viscosity curve as a function of shear rate for the GO sample at different concentrations (0.125–0.5 mg/mL) and temperatures of 37 °C can be seen in Figure 6. The samples exhibit the behavior of Newtonian flow. When the shear rate values are increased, there is a slight increase in the viscosity values. With liquids that have viscosity values of less than 3–4 mPa·s, is not a rotational rheometry appropriate because, at high shear rates, there is a strong effect of inertia and wall slippage that results in an apparent but false increase in viscosity in the high shear region. This effect was observed in previous studies by Cerpa et al. and Carnicer et al. [20,27], where the Newtonian behavior of these types of dispersions was also determined by a microfluidic rheometer at higher shear rates; thus, the apparent shear thickening obtained with rotational rheometry is an artifact of the measurement, because the values are within the detection limit of the apparatus and not real behavior [20,27].

At low concentrations in the range of 0.125–0.5 mg/mL, the GO sample does not modify the viscosity of the biological medium. The mean value of the viscosity, 1.1 mPa·s, is close to the water viscosity, 1 mPa·s. At low concentrations in the range of 0.125–0.5 mg/mL, the GO sample does not modify the viscosity of the biological medium. The mean value of the viscosity, 1.1 mPa·s, is close to the water viscosity, 1 mPa·s. An increase in concentration has little effect at the tested ranges, and the viscosity practically does not change. A value of 1.1 mPa·s for the mean shear rate = 500 s^−1^ was obtained for the GO aqueous dispersion at 37 °C.

**Figure 7:**
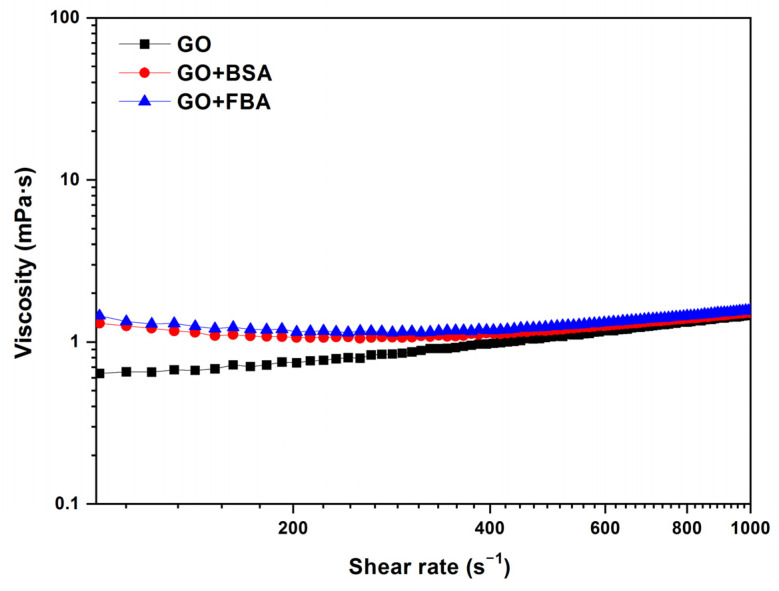
Viscosity (η) vs. shear rate ($\dot{\gamma }$) of GO, GO + BSA, and GO + FBS (GO = 0.25 mg/mL; BSA = 0.02 mg/mL and FBS = 0.2 mg/mL). Temperature: 37 °C.

The effect of the biological fluid is shown in Figure 7. This work compares the GO samples with BSA and FBS, using GO at 0.25 mg/mL, BSA at 0.02 mg/mL, and FBS 0.2 mg/mL, all at 37 °C. The viscosity curve of the GO + BSA and GO + FBS shows a viscosity value higher than for GO dispersions between 0 and 500 s^−1^ due to the interactions and aggregation that can occur between the GO, BSA, and other components of the biological medium. The viscosity value is 1.3 mPa·s for GO + BSA and GO + FBS samples and 0.7 mPa·s for GO at 200 s^−1^. The mean viscosity values of 1.1 mPa·s, 1.2 mPa·s, and 1.25 mPa·s for GO, GO + BSA, and GO + FBS, respectively, at 500 s^−1^, are obtained.

**Figure 8:**
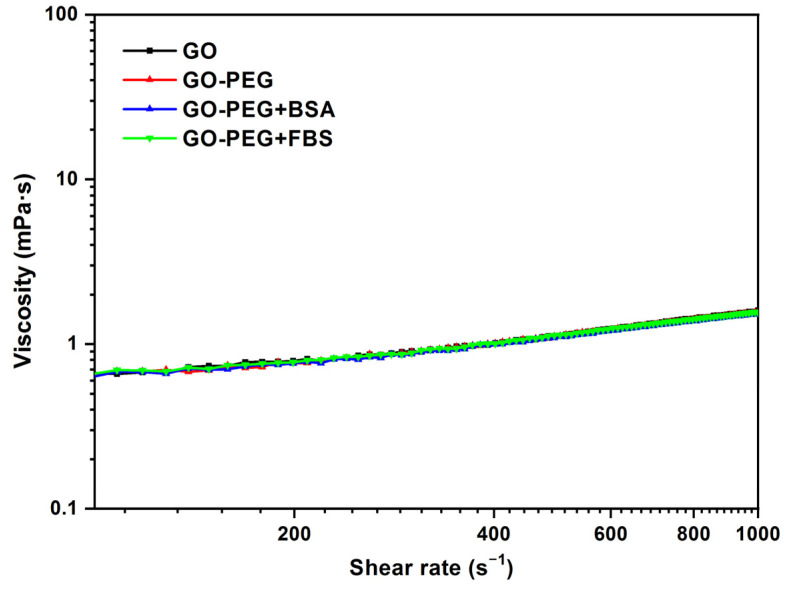
Viscosity (η) vs. shear rate ($\dot{\gamma }$) for 0.125 mg/mL dispersions of GO, GO-PEG, GO-PEG + BSA, and GO-PEG + FBS. Temperature: 37 °C.

The same comparison of viscosity curves was also performed for GO-PEG (Figure 8), using in this case the data for GO 0.125 mg/mL, GO-PEG 0.125 mg/mL, BSA 0.02 mg/mL, and FBS 0.2 mg/mL at 37 °C. Similar results to the previous ones were obtained. The addition of PEG to graphene oxide does not modify the rheological behavior of the dispersion. The addition of PEG helps to stabilize the dispersions and increases the biocompatibility of the nanomaterial, but it does not seem to modify the viscosity values. The viscosity value obtained was 1.1 mPa·s at 500 s^−1^.

**Figure 9:**
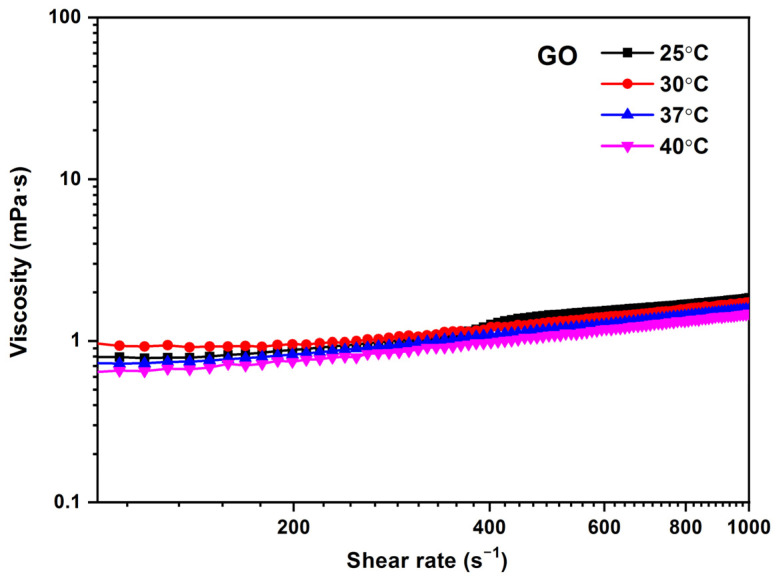
Viscosity (η) vs. shear rate ($\dot{\gamma }$) for different temperatures of GO (0.25 mg/mL). Temperature: 37 °C.

In Figure 9, the influence of temperature on viscosity for the GO 0.25 mg/mL sample is observed as a function of shear rate. It is possible to appreciate that the viscosity values decrease as temperature values increase. At higher temperatures, the viscosity decreases because of the reduction of intermolecular forces due to the thermal energy of the molecule, which increases with increasing intermolecular distances. The viscosity values obtained for GO are between 1.5 and 1.1 mPa·s in the temperature range of 25–40 °C, respectively.

**Figure 10:**
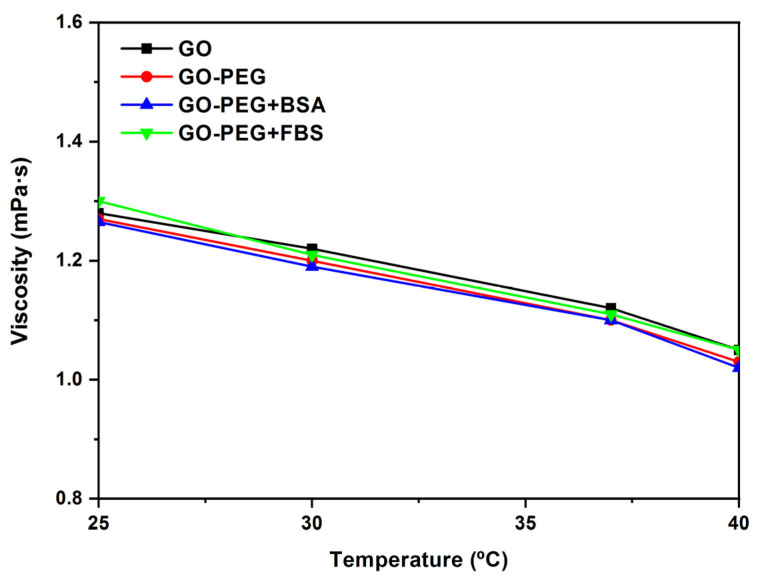
Viscosity (η) vs. temperature (°C) of GO, GO-PEG, GO-PEG + BSA, and GO-PEG + FBS dispersions at a concentration of 0.125 mg/mL and shear rate = 500 s^−1^.

In Figure 10, the viscosity vs. temperature of GO, GO-PEG, GO-PEG + BSA, and GO-PEG + FBS dispersions at a concentration of 0.125 mg/mL and shear rate = 500 s^−1^ are shown. When the temperature increases, the viscosity values decrease. These values are between 1.3 and 1.1 mPa·s at a shear rate = 500 s^−1^ for all samples studied.

## 4. Conclusions

The use and manipulation of materials at the nanometric level has proven to be a very useful tool and presents a wide range of possibilities. Graphene-based nanomaterials have very useful capabilities, one of the most important being the treatment of complicated diseases such as cancer. Therefore, it is essential to know how they behave in contact with biological materials.

The visualization of the samples by SEM has shown that there is a change in the surface of the nanomaterials, with the formation of the corona protein being observed.

Ultraviolet-visible spectrophotometry has revealed that when GO and GO-PEG come into contact with BSA, they form a complex. This indicates that protein binding to the nanomaterial is formed, showing higher absorbance at higher protein concentrations.

A decrease in absorbance could be observed after 60 min of incubation. It is possible to hypothesize that this is the time when the protein desorbs from the nanomaterial.

Further, it has been observed that the incorporation of PEG helps to stabilize the dispersions and increases the biocompatibility of the nanomaterial but does not lead to an increase in protein adsorption.

Rheological characterization has shown that the starting samples exhibit flow behavior characteristic of Newtonian fluids with viscosity values around 1.1 mPa·s at 37 °C temperature.

The PEGylated samples in the presence of BSA do not show significant changes in the viscosity values compared with the starting materials.

As the nanomaterial concentration increases, the viscosity also increases, but the changes are small over the range of concentrations studied for samples (GO and GO-PEG) in the presence of BSA. For the dispersions studied, it is shown that increasing the temperature decreases the viscosity. Viscosity values between 1.5 mPa·s and 1.1 mPa·s are obtained for GO and GO-PEG at a range of temperatures of 25 and 40 °C, and the shear rate = 500 s^−1^.

The use of these graphene nanomaterials should be approached from a sustainable point of view. The preliminary study carried out has shown that they do not present a great risk to the environment, although more exhaustive environmental impact studies should be carried out to ensure responsible use of the nanomaterials.

Looking toward the future, this work can serve as a basis for further studies on the characterization of graphene-based nanomaterials and their behavior in biological media. As described above, they have highly desirable properties for use in biomedicine, such as cancer treatment, biosensors, etc. But they have features that make them dangerous if not used within standardized parameters. They have a very promising future, so it is essential to understand them.

## Figures and Tables

**Table 1:** Concentrations of the dispersions prepared.

Nanomaterial	BSA (mg/mL)			
GO 0.25 mg/mL	0.01	0.02	0.03	0.04
GO-PEG 0.125 mg/mL	0.01	0.02	0.03	0.04

**Table 2:** Zeta potential measurement of GO suspended in BSA at 0.01 mg/mL and 25 °C.

pH	Zeta Potential (mV) GO + BSA
2	35.5
3	33.6
4	17.7
5	−3.8
6	−15.0
7	−29.5
8	−33.7
9	−36.4

## Data Availability

Not applicable.
